# SuperSAGE analysis of the *Nicotiana attenuata *transcriptome after fatty acid-amino acid elicitation (FAC): identification of early mediators of insect responses

**DOI:** 10.1186/1471-2229-10-66

**Published:** 2010-04-14

**Authors:** Paola A Gilardoni, Stefan Schuck, Ruth Jüngling, Björn Rotter, Ian T Baldwin, Gustavo Bonaventure

**Affiliations:** 1 Max Planck Institute for Chemical Ecology, Department of Molecular Ecology, Hans Knöll Str. 8, 07745 Jena, Germany; 2 GenXPro GmbH, Altenhöferallee 3, 60438 Frankfurt am Main, Germany

## Abstract

**Background:**

Plants trigger and tailor defense responses after perception of the oral secretions (OS) of attacking specialist lepidopteran larvae. Fatty acid-amino acid conjugates (FACs) in the OS of the *Manduca sexta *larvae are necessary and sufficient to elicit the herbivory-specific responses in *Nicotiana attenuata*, an annual wild tobacco species. How FACs are perceived and activate signal transduction mechanisms is unknown.

**Results:**

We used SuperSAGE combined with 454 sequencing to quantify the early transcriptional changes elicited by the FAC *N*-linolenoyl-glutamic acid (18:3-Glu) and virus induced gene silencing (VIGS) to examine the function of candidate genes in the *M. sexta*-*N. attenuata *interaction. The analysis targeted mRNAs encoding regulatory components: rare transcripts with very rapid FAC-elicited kinetics (increases within 60 and declines within 120 min). From 12,744 unique Tag sequences identified (UniTags), 430 and 117 were significantly up- and down-regulated ≥ 2.5-fold, respectively, after 18:3-Glu elicitation compared to wounding. Based on gene ontology classification, more than 25% of the annotated UniTags corresponded to putative regulatory components, including 30 transcriptional regulators and 22 protein kinases. Quantitative PCR analysis was used to analyze the FAC-dependent regulation of a subset of 27 of these UniTags and for most of them a rapid and transient induction was confirmed. Six FAC-regulated genes were functionally characterized by VIGS and two, a putative lipid phosphate phosphatase (LPP) and a protein of unknown function, were identified as important mediators of the *M. sexta*-*N. attenuata *interaction.

**Conclusions:**

The analysis of the early changes in the transcriptome of *N. attenuata *after FAC elicitation using SuperSAGE/454 has identified regulatory genes involved in insect-specific mediated responses in plants. Moreover, it has provided a foundation for the identification of additional novel regulators associated with this process.

## Background

*Nicotiana attenuata *is an annual native to Southwestern USA that germinates from seed banks in response to factors in wood smoke after fires [1]. Because of this germination behavior and a strong intra-specific competition, *N. attenuata *allocates resources primarily to sustain rapid growth and seed setting and as a consequence, it has developed a large number of induced defense responses to ward off the unpredictable attacks from herbivores [2]. Hence, when *N. attenuata *is attacked by insect folivores, an extensive reprogramming of its transcriptome, proteome and metabolome takes place [3-5]. Previous studies estimated that more than 500 *N. attenuata *genes respond to *Manduca sexta *larval feeding [6] and demonstrated that the plant readjusts its metabolism for *de novo *synthesis of direct and indirect defense responses and to induce tolerance mechanisms [7-9]. Activation of these defensive mechanisms requires energy and resources from primary metabolism and involves therefore a complex rearrangement of resource allocation in the plant, including altered photosynthesis and sink/source relations [5]. How plants decode insect feeding and trigger defense and tolerance responses is starting to be understood. For example, a SnRK1 kinase complex has been found to regulate tolerance mechanisms associated to the leaf/root partition of photoassimilates [9] and two MAPKs, WIPK and SIPK (Wound-induced and Salicylate-Induced Protein Kinases, respectively), were shown to be critical for the induction of direct defense responses in *N. attenuata *[10].

Herbivore attack induces in plants the coordinated activation of several signal cascades including those of jasmonic acid (JA), salicylic acid (SA), and ethylene (ET) [11]. Among them, JA plays a major and essential role in the induction of a large number of the plant's protective responses against insect herbivory and wounding [12,13]. Thus, having JA as a common signal, a large number of the plant responses to these two stimuli overlap, however, plants can differentiate between mechanical damage and insect herbivory to tailor their responses. The perception of components in the oral secretions (OS) of feeding larvae is one mechanism by which plants can decode insect feeding. Fatty acid-amino acid conjugates (FAC) are major components in the OS of *M. sexta *larvae and they are necessary and sufficient to induce most of the defense responses triggered by feeding *M. sexta *caterpillar in *N. attenuata *[14]. Hence, previous studies suggest the existence of central herbivore-activated regulators in *N. attenuata *leaves, which, in turn, are regulated by minute amounts of FACs in the insect's OS. Disentangling the effect of mechanical tissue damage and FAC elicitation will provide critical information on how plants control changes in its metabolism to more efficiently reduce the negative fitness consequences of herbivore attack.

One of the earliest known molecular events differentially induced by OS and FACs in tobacco is the activation of WIPK and SIPK. Activation of these protein kinases occur within the first minutes after wounding [15] and the activation is enhanced several-fold by applying *M. sexta *OS to wounds [10]. Importantly, not only their activities but also SIPK and WIPK transcript levels are rapidly (within 60 min) and transiently induced after elicitation [10,15], indicating that these regulators are under positive feedback control at the transcriptional level. One of the early targets of the FAC signal transduction pathway in *N. attenuata *is the *WRKY6 *gene. Its transcript levels are also rapidly and transiently induced after wounds have been supplemented with *M. sexta *OS or synthetic FACs but only marginally by mechanical damage alone [16]. This rapid and transient kinetic of mRNA accumulation is characteristic of regulatory components and differs from that showed by, for example, transcripts encoding for defense components (e.g., protease inhibitors) which is characterized by a slower and more persistent rate of mRNA accumulation, reaching maximum levels after hours to days [8]. However, not all regulatory components are under positive feedback control at the transcriptional level: the *Coronatine Insensitive 1 *(*COI1*) gene is an example in the JA transduction pathway [17]. However, some of the recently identified JAZ proteins that interact with COI1 and participate in JA-Ile perception are rapidly and transiently induced at the mRNA level by wounding in Arabidopsis [18].

The rapid advances in high throughput sequencing capacity in combination with new "open-architecture" techniques for quantification of gene expression has opened the possibility of performing genome-wide transcriptome studies in organisms from which massive nucleotide sequence information is not yet available. Serial analysis of gene expression (SAGE) is a technique that allows for the absolute quantification of mRNA abundance by quantifying the relative frequencies of individual short (13 nt) transcripts signatures tags [19]. Further development of the technique allowed for the generation of 26 nt tags (SuperSAGE) [20] which substantially improved the annotation of tags when aligned to sequences in public nucleotide databases [21]. With these techniques, the detection of transcripts is proportionally correlated to the scale of DNA sequencing and their combination with next generation sequencing (NGS) allows for the detection and analysis of very low abundant transcripts (frequently encoding for regulatory components) which have been estimated to account for more than 90% of mRNAs in eukaryotic cells [20,22].

Here we used SuperSAGE in combination with NGS for the quantification of the early changes (within 30 min) occurring in the transcriptome of *N. attenuata *plants after a single event of 18:3-Glu elicitation. The major objective of the study was to identify genes encoding for potential regulatory components of the FAC-mediated responses by looking for low abundant transcripts that were rapidly and transiently induced after 18:3-Glu elicitation.

## Results

### Generation of SuperSAGE libraries from wounded and FAC elicited *N. attenuata *leaves

**Figure 1:**
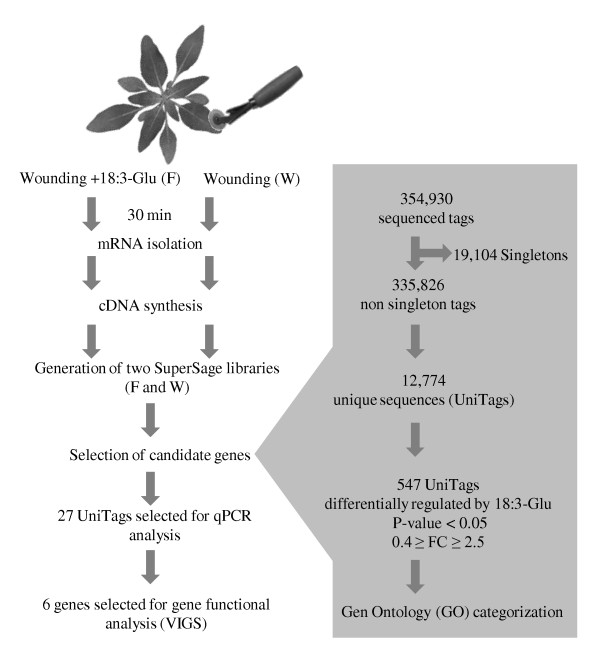
**Schematic representation of the approach used for identification of regulatory genes by SuperSAGE**. Two SuperSAGE libraries were generated from the second fully expanded leaf of *N. attenuata*. Plants were mechanically wounded or wounded plus the immediate addition of 18:3-Glu as a single FAC elicitor and leaves were harvested 30 min after the treatments. From these libraries, 547 unique mRNA sequences (UniTags) were defined as differentially expressed after 18:3-Glu elicitation versus wounding (FC: fold-change). After gene ontology categorization, the kinetics of transcript accumulation corresponding to 27 UniTags were analyzed by quantitative PCR. Six selected genes were functionally characterized by Virus Induce Gene Silencing (VIGS).

Two SuperSAGE libraries were generated from the second fully expanded leaf of *N. attenuata *plants either mechanically wounded or wounded and supplemented with 18:3-Glu as a single FAC elicitor. Leaf samples were harvested after 30 min of the treatments (Figure 1). Wounding is a prerequisite for FAC-elicitation; hence, analysis of wounded leaves was used to differentiate between genes regulated by mechanical damage from those regulated more specifically or deferentially by FACs. Additionally, elicitation by a single elicitor (18:3-Glu) and a single wound event were used to eliminate the effects of other OS components and repeated wounding on gene expression.

The total number of SuperSAGE tags obtained after sequencing the libraries in a single 454 plate and eliminating i) incomplete reads, ii) twin-ditags, and iii) ditags without complete library-identification DNA linkers was 354,930; comprising 227,536 tags from the wounding (W) library and 127,394 from the FAC-elicited (F) library (Table 1). These tags represented 31,878 unique sequences with 19,104 (11,951 in the W library and 7,153 in the F library) detected only once (singletons) in the combined libraries, and 12,774 detected at least twice in the combined libraries (Table 1). These latter tags are referred as UniTags throughout the manuscript [22] and will be considered for further analysis. Singletons represented thus ~60% of unique sequences, in agreement with previous studies [21,22]. The complete SuperSAGE dataset is available in Additional file 1 (see also Accession numbers).

**Table 1:** Features of the SuperSAGE libraries from wounded and 18:3-Glu elicited leaves

Library	W**	F**	Total	(%)
Sequenced tags	227,536	127,394	354,930	(100)
Number of unique transcripts (UniTags)	11,942	10,117	12,774	
Number of singletons	11,951	7,153	19,104	

Abundance classes of UniTags*				
Very high-abundant: > 5,000 copies.million^-1^	24	22	46	(0.2)
High-abundant: > 1,000 - 5,000 copies.million^-1^	127	133	260	(1.2)
Mid-abundant: 100 - 1,000 copies.million^-1^	1,178	1,084	2,262	(10.2)
Low-abundant: < 100 copies.million^-1^	10,613	8,878	19,491	(88.4)
**Total**	11,942	10,117		

Copy number of Tags in Abundance classes*				
Very high-abundant: > 5,000 copies.million^-1^	2.34 × 10^5^	2.34 × 10^5^	4.68 × 10^5^	(23.4)
High-abundant: > 1,000 - 5,000 copies.million^-1^	2.36 × 10^5^	2.43 × 10^5^	4.80 × 10^5^	(24.0)
Mid-abundant: 100 - 1,000 copies.million^-1^	3.32 × 10^5^	3.16 × 10^5^	6.48 × 10^5^	(32.4)
Low-abundant: < 100 copies.million^-1^	1.98 × 10^5^	2.07 × 10^5^	4.05 × 10^5^	(20.2)
**Total**	1.00 × 10^6^	1.00 × 10^6^		

* Values normalized to 1 million tags

** W: wounded; F: 18:3-Glu elicited

### Abundance of UniTags and annotation to public databases

The UniTags were first classified in abundance groups according to their number of copies [22]. UniTags present at ≤ 100, > 100 - ≤ 1,000, > 1,000 - ≤ 5,000 and >5,000 copies per million (copies.million^-1^) were considered as low-, mid-, high- and very high-abundant tags, respectively (Table 1). The frequency distribution of the 12,774 UniTags showed that the number of copies in low and mid abundance groups (≤ 1,000 copies.million^-1^) represented > 98% of the UniTags while high- and very high-abundant tags (>1,000 copies.million^-1^) represented only 1.4% (Table 1). However, although the latter group represented only a small fraction of the 12,774 UniTags, together they accounted for ~47% of the total number of tag copies in both the W and F libraries (Table 1). These values were in agreement with previously reported data [23,24].

Annotation of the 12,774 UniTags using basic local alignments (BLASTN) gave 5,565 tags (43.6%) that matched with a maximum of 3 mismatches (score ≥ 46.1 or e-value ≤ 6.10^-4^) to sequences deposited in GenBank plant nucleotide databases (Additional file 1 and Table 2). 78.8% of these 5,565 UniTags matched perfectly (26/26) with sequences in the databases while 8.4% did it with one mismatch (25/26), 6.5% with two mismatches (24/26) and 6.4% with three mismatches (23/26; Table 2). Moreover, 88% of the annotated UniTags matched sequences corresponding to *Nicotiana *spp, 5% to *Solanum *spp and 8% to other plant species (Table 2).

**Table 2:** Annotation of UniTags using GenBank DNA sequence databases

	No. of matches (total 26)					
	**26**	**25**	**24**	**23**	**Total**	**(%)**

*Nicotiana *spp	3,867	403	305	300	**4,875**	(88)
*Solanum *ssp	188	31	19	22	**260**	(5)
Other species	330	32	35	33	**430**	(8)

Total	**4,385**	**466**	**359**	**355**	**5,565**	(100)
(%)	(78.8)	(8.4)	(6.5)	(6.4)		

### FAC elicitation induces differential expression of 547 UniTags

**Figure 2:**
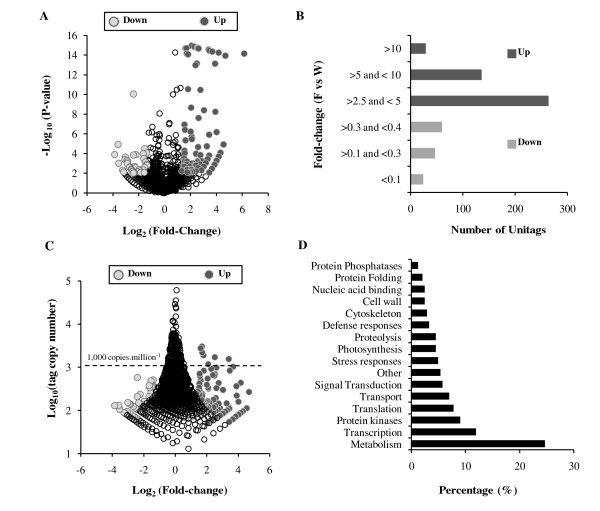
**Analysis of differentially expressed UniTags**. **A**, Volcano plot showing the Log_2_(fold-change; F vs. W) versus Log_10_(*P*-value) of 547 expressed UniTags. **B**, Fold change (F vs. W) distribution of the 547 differentially expressed UniTags. **C**, Distribution of the expressed UniTags based on the Log_2_(Fold-change; F vs. W) versus Log_10_(tag copy number). The dashed line corresponds to a threshold of 1,000 copies.million^-1^. **D**, Distribution of 242 annotated UniTags in Gene Ontologiy (GO) categories based on Molecular Function and Biological Process.

Statistically significant changes in tag copy number between the F and W libraries were analyzed by calculating a probability (*P*)-value according to [25] (see Materials and Methods for a brief description). Although small changes in expression levels may have biological significance [25], in this study we focused primarily on genes which showed strong changes in expression levels with arbitrary fold-change (FC) values ≥ 2.5 or ≤ 0.4 (FAC elicitation vs wounding). Based on the calculated (*P*)-values and using a 95% confidence level, 547 UniTags were identified as differentially expressed after FAC elicitation (Additional file 2). Among these UniTags, 430 had FC ≥ 2.5 and 117 FC ≤ 0.4 (F vs W; Figure 2a and Additional file 2). Most of the differentially expressed UniTags presented FC values between 0.2 and 10, with 29 and 24 UniTags presenting FC values ≥ 10 and ≤ 0.2, respectively (Figure 2b). The majority (98.6%) of the differentially up-regulated UniTags and all of the down-regulated UniTags corresponded to low- and mid-abundance groups (< 1,000 copies.million^-1^; Figure 2c and Additional file 2), indicating that the strongest changes in expression levels occurred primarily in genes expressed at low to intermediate levels.

### Assignment of differentially expressed UniTags to Gene Ontology (GO): biological and functional categories

To obtain gene function categories of the differentially expressed UniTags, gene ontology (GO) annotation was performed by BLASTX (using the corresponding annotated nucleotide sequences as queries) against the non-redundant GenBank and UniProtKB/TrEMBL protein databases (Additional file 2). For this analysis, we used UniTags that showed a maximum of 2 mismatches (24/26) with entries in the GenBank nucleotide database (Additional file 1). Of the 547 differentially expressed UniTags, 349 had an associated nucleotide sequence and 323 matched to an amino acid sequence entry (e-value < 9.10^-4^) in the GenBank and UniProtKB/TrEMBL databases (Additional file 2). GO annotations (biological processes and/or molecular function) could be assigned to 242 of these 323 UniTags with the remaining entries corresponding to uncharacterized proteins (Additional file 2).

Among the most prevalent GO biological processes, ~25% of the UniTags classified into metabolism, ~12% into regulation of gene expression (including transcription, nucleosome assembly and mRNA processing), ~10% into amino acid phosphorylation/dephosphorylation, ~8% into translation (including ribosome assembling), ~8% into defense and stress responses, ~7% into transport, ~6% into protein degradation and folding and ~6% into signal transduction components (Figure 2d). The preponderance of changes in transcripts corresponding to metabolism, signaling, transcription, translation and transport associated processes after 30 min of 18:3-Glu elicitation emphasized the fact that at this early time point a substantial reprogramming of the leaf metabolism is already in progress. Based on changes in metabolic genes, hallmarks of this reprogramming included an increased capacity for protein synthesis and the generation of C skeletons and reducing power (see Discussion). These changes in the expression of metabolic genes are consistent with a substantial shift in primary metabolism to support secondary metabolism and tolerance mechanisms [5] and are consistent with previous gene expression studies [3,6,26] (see Discussion). The identification of regulatory factors controlling these changes in metabolism and defense and tolerance processes against insects is one of the major challenges for the future and some potential candidates are described below.

### Changes in the expression of UniTags/mRNAs encoding for regulatory components

The most prevalent GO molecular function with regulatory activity corresponded to transcriptional regulators and protein kinases, represented by 30 and 22 UniTags, respectively. The protein phosphatase category contained 3 UniTags, the signal transduction category 14 UniTags and the nucleic acid binding category 6 UniTags (Additional file 2). Thus, a total of 75 annotated UniTags corresponded to factors with potential regulatory function.

Among transcriptional regulators, UniTags corresponding to WRKY transcription factors (TFs) were the most predominant (seven UniTags) and Tag-995 was the most up-regulated (23 fold) after 18:3-Glu elicitation within this group. Other UniTags for WRKYs were up-regulated between 9 and 2.5 fold (Additional file 2). Within the WRKY domain containing family, a WIZZ TF (wound-induced leucine *z*ipper *z*inc finger) [27] was up-regulated 7 fold. Other prevalent up-regulated TFs included AP2-like factors (three UniTags; up-regulated between 9 and 3 fold), RAV factors (two UniTags; up-regulated ~3 fold), ethylene-responsive element binding proteins (EREBP; two UniTags; up-regulated between 9 and 3 fold) and CCR4-NOT transcription complex proteins (two UniTags; up-regulated between 7 and 3 fold) (Additional file 2). Single up-regulated UniTags in this category corresponded to a bZIP TF (2.5 fold), HIS4 (2.5 fold), S1FA (7 fold), RNA polymerase II (RNAPII; 5.5 fold) and a sigma subunit for a plastidial RNA polymerase (7 fold). Among down-regulated transcriptional regulators were a GATA-1 zinc finger protein and RNA polymerase III (RNAPIII; Additional file 2).

Within the protein kinase and phosphatase classes, three UniTags corresponded to MAPK (two up-regulated between 4 and 2.5 fold and one down-regulated 10 fold), three to cell-wall associated kinases (WAK; up-regulated between 3.5 and 6 fold), two to BRASSINOSTEROID INSENSITIVE 1-associated receptor kinase 1 (BAK1; up-regulated between 9 and 3 fold) and three to protein phosphatase 2A (PP2A) and C (PP2C; two up-regulated ~3 fold and one down-regulated ~5-fold). In addition, this category contained a chloroplast precursor for *Arabidopsis *protein kinase 1 (APK1) [28] up-regulated ~7 fold, a shaggy-like kinase (up-regulated ~5 fold), and a cytokinin-regulated kinase 1 (CRK1; the most up-regulated, ~14 fold) and a calmodulin protein kinase 1 (up-regulated ~11 fold) among others (Additional file 2).

Within the signal transduction class, the most predominant UniTags corresponded to "Avr9/Cf-9 rapidly elicited proteins" (seven UniTags) up-regulated between 13 and 2.5 fold. Single up-regulated UniTags corresponded to a Hs1^pro-1^-like protein (putative nematode resistance protein (NRP); 17.9 fold), SGT1 (3.6 fold), a lipid phosphate phosphatase (LPP; 5.4 fold) and an extra-large G protein (2.5 fold) were also contained in this category (Additional file 2).

### Validation of the SuperSAGE data by qPCR

A subset of 27 differentially expressed UniTags (Table 3) was selected for further analysis based on the fulfillment of at least two of the following criteria: 1) strong and significant changes in their FC values (either up- or down-regulated, F vs W); 2) abundance of <1,000 copies.million^-1 ^(as regulatory components are encoded by low abundant transcripts); 3) matched known regulatory components in the databases.

**Table 3:** List of the 27 UniTags selected for qPCR and VIGS analysis^1^

Tag-Id	Tag sequence	FC	Protein Description
Tag-11166	CATGTGTCAAGCTGGAAAACTTGCCA	69.92	NM
Tag-4898	CATGCTGCTGGGACTCTCGTATACAG	25.78	NM
Tag-995	CATGAATTCAAGAAACAAGCCAACAA	23.31	ACJ04728.1| WRKY transcription factor
Tag-6642	CATGGCCAAGAGTACGTTCTCAAAGG	19.72	AAL08561.1| auxin-regulated protein
Tag-895	CATGAATGACACTAATGAATTCGTCG	19.72	NM
**Tag-6205**	**CATGGATCTACGCGTCAAAAATGCTT**	**17.93**	**AAG44839.1| Hs1pro-1-like receptor**
Tag-2452	CATGATGAATACGAGCAGCTTCGGGT	17.93	NM
Tag-1439	CATGACTGCTGTCAGACGAACTGCAC	16.14	BAD33355.1| ABC transporter
**Tag-837**	**CATGAATCATCCAATATGGTATGGGC**	**14.70**	**XP_002298932.1| predicted protein (UnkA)**
Tag-9719	CATGTATTCTGCTGTAAATTCAGGAA	12.77	AAG43557.1| Avr9/Cf-9 rapidly elicited protein
Tag-2978	CATGATTTTTTTTCCTTCTGCTGTAT	12.55	NM
**Tag-12314**	**CATGTTTAGAGCAATGAGTACACGAA**	**10.81**	**EEF40825.1| hypothetical protein (UnkB)**
Tag-6199	CATGGATCGGCAAACAAAGAGATTAT	10.51	NM
Tag-7795	CATGGGTTATTCAGTGCTGTTCAGTG	5.98	AAY17949.1| ring zinc finger protein
Tag-1844	CATGAGGAAGGCTATGAAGGAGAAGA	5.82	NM
Tag-7036	CATGGCTGCTGACAACTTACCTGGAT	5.79	ACG41445.1| plastid-lipid associated protein
**Tag-11559**	**CATGTTATCAGTTAACTAATAAAAGC**	**5.70**	**EEF35389.1| wall-associated kinase (WAK)**
**Tag-10039**	**CATGTCCACCATACTAACGGAGGATT**	**5.38**	**NP_001078095.1| LPP (Lipid Phosphate Phosphatase)**
Tag-6032	CATGGAGGTCTTTCTCGTTATCTGAT	5.22	XP_002278077.1| hypothetical protein
Tag-5869	CATGGAGACTTTGCAAGTTAAGTTTT	4.26	BAC07504.2| receptor-like protein kinase
Tag-2067	CATGAGTTGGTGGATTCAAATCTTGG	4.13	EEF37528.1| wall-associated kinase (WAK)
Tag-129	CATGAAACACAGTTAGCAATTTATGA	4.03	ABD28351.1| Lissencephaly type-1-like homology
Tag-6938	CATGGCTCGGATTTGCATCTCTAAAG	3.84	NP_563839.1| transcription factor
Tag-5283	CATGCTTTGTAAAACTTAGCAACAAA	3.47	NM
**Tag-2815**	**CATGATTGAGTTGCAAAGCAGTGGAG**	**3.36**	**BAB16427.1|*Nicotiana *Elicited Induce Gene (NEIG)**
Tag-9434	CATGTATAGCAGATTGGTGAAATGAT	3.19	BAE44121.1| protein phosphatase 2C
Tag-2990	CATGCAAAACGTACACCGAGAAAGAA	0.09	NM

FC: fold change (FAC-elicitation vs. Wounding). NM: no match in GenBank

^1^UniTags selected for VIGS analysis are depicted in bold.

The selected UniTags were first elongated by amplification of their corresponding cDNAs and BLASTed against the GenBank plant nucleotide databases to confirm their identities. All of the elongated sequences (see "Accession numbers") matched to the same entries as the original 26 bp tags (data not shown). Secondly, the elongated sequences were used to design gene-specific primers to i) validate the SuperSAGE data and ii) to study the kinetic of mRNA induction by real time quantitative PCR (qPCR). Total RNA was extracted from both wounded and 18:3-Glu elicited leaves of WT plants after different times of the stimuli.

**Figure 3:**
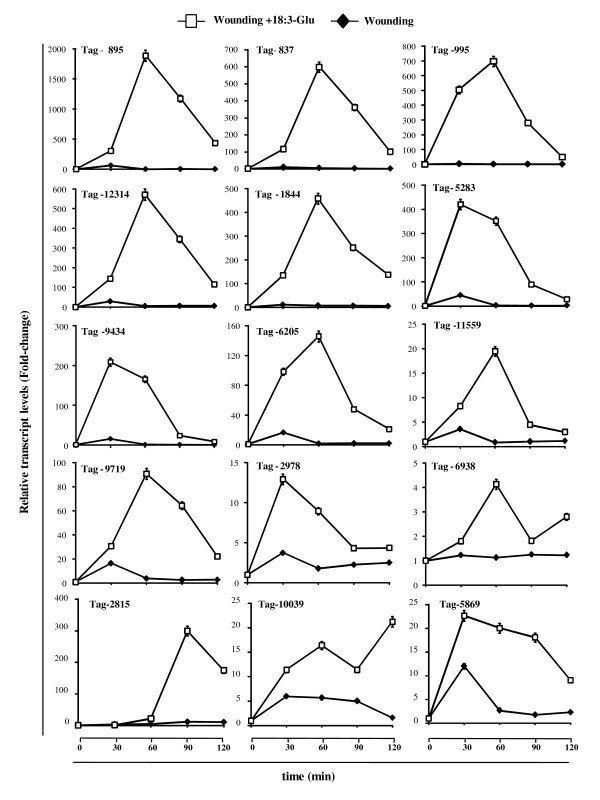
**Analysis of mRNA accumulation corresponding to selected UniTags by qPCR**. Examples of the kinetics of induction of mRNAs for 15 UniTags analyzed by qPCR after wounding and 18:3-Glu elicitation. Relative mRNA quantification was performed using the eEF1A as a reference gene for normalization and the data is expressed as fold-change relative to time 0 (unelicited leaves). Values at this time point were set arbitrary to 1. Transcripts levels were analyzed in three biological replicates (*n *= 3).

The accumulation of 20 mRNAs corresponding to the selected UniTags was consistent with a rapid increase (within 60 min) after FAC elicitation and a rapid decrease (within 120 min) to basal or lower levels after the stimuli (Figure 3 and Additional file 3 [Figure S1]). Interestingly, several transcripts showed either no or minimal induction by wounding, representing therefore genes activated almost specifically by FACs (e.g., 837, 995, 1844, 2815; Figure 3). For some transcripts mechanical damage induced an increase in their corresponding mRNA levels which was potentiated several fold by 18:3-Glu elicitation (e.g., 5869, 10039; Figure 3). For transcripts corresponding to four UniTags (6032, 7036, 129, 6642), the differential regulation by 18:3-Glu elicitation could not be confirmed (Additional file 3 [Figure S1]) and they may represent false positives in the SuperSAGE analysis [25]. Finally, mRNAs for three UniTags (1439, 2452, 2990) were differentially repressed by 18:3-Glu elicitation (Additional file 3 [Figure S1]).

### Functional characterization of candidate regulatory components of insect mediated responses by VIGS

To validate the use of the SuperSAGE approach for the identification of candidate regulatory components of the interaction between *N. attenuata *and *M. sexta *larvae, six genes were selected for preliminary gene function characterization by virus-induced gene silencing (VIGS). The selection of these genes was based on: 1) their kinetic of mRNA induction and 2) their fold-change compared to wounding (minimal induction by wounding -except for Tag-10039). Some of the genes encoded for putative regulatory components and two presented no similarity to any other protein of known or predicted function (Figure 3 and Table 3). The selected UniTags corresponded to a Hs1^pro-1^-like protein (putative nematode resistance protein (NRP); Tag-6205), lipid phosphate phosphatase (LPP; Tag-10039), *Nicotiana *elicitor induced gene (NEIG; Tag-2815), cell wall-associated protein kinase (WAK; Tag-11559), UnkA (Tag-837) and UnkB (Tag-12314) (these last two presenting no protein annotation) (Table 4). To evaluate whether these genes participate in FAC- and insect defense-mediated responses, gene-specific silenced plants and plants transformed with the empty vector (EV; control plants) were assessed for *M. sexta *larval performance and the accumulation of JA and JA-Ile after 18:3-Glu elicitation and wounding. Gene silencing efficiency in these plants was analyzed by qPCR in 18:3-Glu-elicited leaves after 1 h of the treatment (Table 4). The morphological phenotype of the silenced-plants was indistinguishable from EV control plants (data not shown).

**Table 4:** Selected genes for functional characterization by VIGS

Tag ID	Gene Name	VIGS construct	Silencing efficiency (%)^1^
Tag-6205	Nematode Resistance Protein (NRP)	pTVNRP	67 ± 1.8
Tag-10039	Lipid Phosphate Phosphatase (LPP)	pTVLPP	69 ± 2.1
Tag-11559	Wall Assocaited Kinase (WAK)	pTVWAK	83 ± 2.5
Tag-2815	*Nicotiana *Elicited Induced Gene (NEIG)	pTVNEIG	91 ± 5.2
Tag-837	UnkA	pTVUNKA	73 ± 4.7
Tag-12314	UnkB	pTVUNKB	98 ± 2.8

^1 ^The silencing efficiency is expressed as the reduction (%) of the mRNA levels in the VIGS-silenced plants relative to the levels of the corresponding mRNA in EV (empty vector) plants. In all cases the reductions were statistically significant (*P *< 0.05, *t*-test, EV vs VIGS).

**Figure 4:**
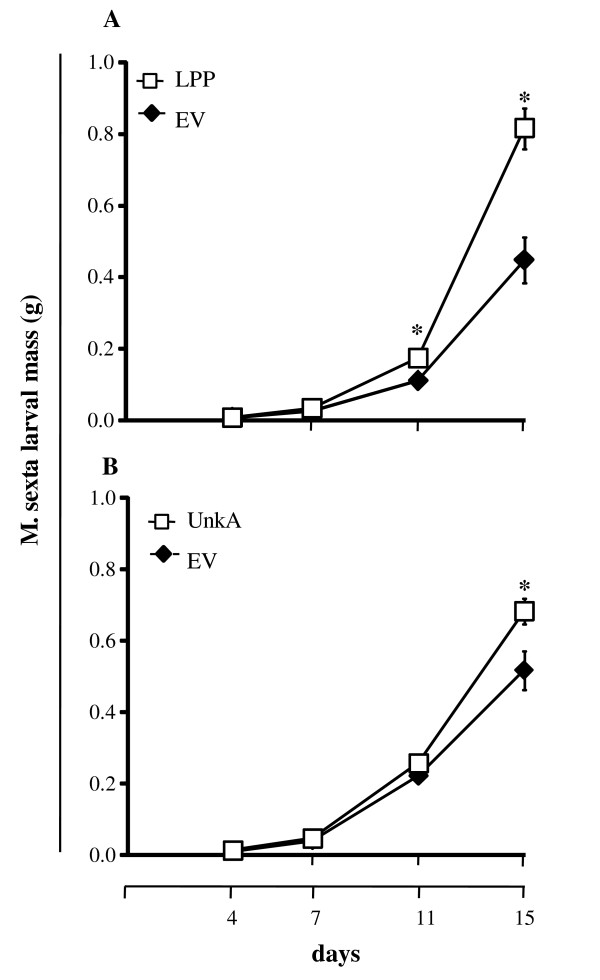
***M. sexta *larval performance on LPP- and UnkA-silenced plants**. *N. attenuata *plants were silenced in the expression of LPP and UnkA by VIGS. Plants transformed with the empty vector (EV) were used as control. **A**, Mean (± SE) of *M. sexta *larval mass after 4, 7, 11 and 15 days of feeding on EV and LPP-silenced plants (*n *= 32 for each genotype). Statistical analysis was performed by repeated-measurement ANOVA (F_1,54 _= 12.79, *P *< 0.01). **B**, Mean (± SE) of *M. sexta *larval mass after 4, 7, 11 and 15 days of feeding on EV and UnkA-silenced plants (*n *= 32 for each genotype). Statistical analysis was performed by repeated-measurement ANOVA (F_1,48 _= 6.62, *P *< 0.05). In both cases asterisks represent significant differences between EV and the corresponding silenced line. Both experiments were conducted two times independently with identical results.

**Figure 5:**
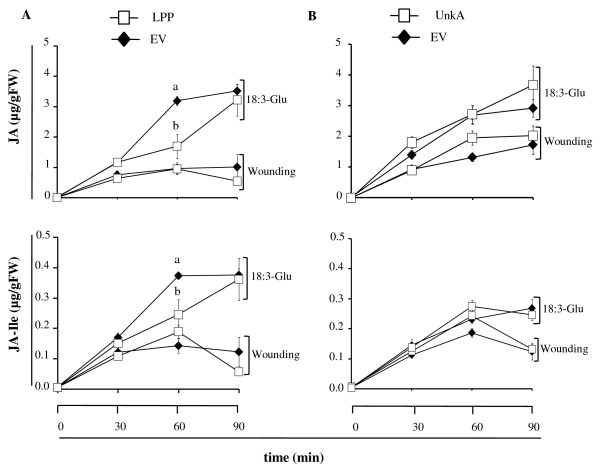
**Quantification of JA and JA-ILE levels in LPP- and UnkA-silenced plants**. Leaves from *N. attenuata *plants silenced in the expression of LPP and UnkA by VIGS were either wounded or 18:3-Glu elicited and tissue was harvested at different times. **A**, Mean (± SE) JA and JA-ILE levels in EV and LPP-silenced plants (*n *= 5). Statistical differences in JA and JA-ILE levels were analyzed by univariate ANOVA (JA, F_3,12 _= 18.6, *P *< 0.001; JA-ILE, F_3,12 _= 5.6, *P *< 0.05). Different letters denote significant differences in a Scheffé *post-hoc *test at *P *< 0.05. **B**, Mean (± SE) JA and JA-ILE levels for EV and UnkA-silenced plants (*n *= 5).

*M. sexta *larva feeding on plants silenced in the expression of LPP or UnkA showed significant increases in mass gained after 11 and/or 15 days compared to EV plants (Figure 4; see caption for statistical analysis). In contrast, larval performance was similar between EV and plants silenced in NRP, NEIG, WAK and UnkB (Additional file 3 [Figure S2]). The rate of JA and JA-Ile accumulation after wounding was similar between EV and LPP-silenced plants (Figure 5a). After 18:3-Glu elicitation, the accumulation of JA and JA-Ile was significantly slower in LPP-silenced plants however after 90 min the levels were similar to EV plants (Figure 5a, see caption for statistical analysis). Plants silenced in UnkA expression had similar rates of JA and JA-Ile accumulation to EV plants after both 18:3-Glu elicitation and wounding (Figure 5b). Likewise, induced levels of JA and JA-Ile in NRP-, WAK-, NEIG- and UnkB-silenced plants were similar to EV plants (data not shown).

## Discussion

In this study we exploited the combined capacities of SuperSAGE and NGS to quantify the expression of thousands of genes in *N. attenuata *leaves elicited by one of the major elicitors (18:3-Glu) present in the OS of *M. sexta *larvae. We analyzed the expression of >335,000 SuperSAGE tags, representing 12,774 unique transcript sequences with the main objective of identifying factors with potential regulatory functions during the *M. sexta*-*N. attenuata *interaction. The analysis disclosed 75 annotated putative regulatory factors and from a subset of 27 selected we could confirm that the kinetic of mRNA induction for 20 of them followed the expected profile, a rapid and transient up-regulation.

Because the SuperSAGE generates 26 nt tags, DNA sequence databases are a prerequisite to warrant efficient gene annotation of the tags. Consistent with the presence of >17,000 *Nicotiana *spp nucleotide sequences publicly available in GenBank, ~88% of the *N. attenuata *UniTags matched to *Nicotiana *species (Table 2). However, only 43.5% of the 12,774 UniTags matched -with a maximum of 3 mismatches- to sequences in GenBank (Table 2). With a tolerance of 6 mismatches (20/26), 8,151 UniTags (64%) found a hit in this database (Additional file 1). Most SuperSAGE tags are derived from the 3' UTR of each mRNA molecule [20] which has been shown to be allele-specific in plants [29]. Since most of the *Nicotiana *spp nucleotide entries in GenBank correspond to *N. tabacum*, a percentage of the mismatches may be attributed to polymorphisms in the 3' UTR of mRNAs from *N. attenuata *and this tobacco species. Regarding the 547 differentially expressed UniTags, 60% could be assigned to a protein entry (Additional file 2) in GenBank and UniProtKB/TrEMBL protein databases and 25% of this fraction represented fully uncharacterized protein entries (Additional file 2), a fact that partially handicapped the functional characterization of the *N. attenuata *transcription profiles. Nevertheless, a total of 242 UniTags were reliably assigned to a GO category. However, since these 242 UniTags represented < 50% of the differentially regulated mRNAs (Additional file 2), we expect that improved gene annotation will increase (probably by factor of two) the number of putative regulators that change expression after 18:3-Glu elicitation.

### Changes in the expression of mRNAs encoding for regulatory components

WRKY transcription factors (TFs) occur in large gene families in plants and orchestrate different responses including those for pathogen resistance and wound healing [30,31]. For example, WRKYs bind to W-box elements in PR1 genes and regulate their expression after salicylic acid (SA) induction and pathogen elicitation [32]. WRKY3 and 6 in *N. attenuata *have been involved in responses against insect herbivores [16]. WIZZ (wound-induced leucine zipper zinc finger) was identified as an early and transiently activated wound-responsive gene in tobacco [27] and contains a leucine-zipper motif and a WRKY domain in its structure. After wounding, WIZZ transcripts accumulate within 10 min reaching maximal levels by 30 min and decreasing thereafter to basal levels [27]. Our results suggested that several WRKY members including WIZZ may play critical roles in the coordination of *M. sexta-N. attenuata *interactions. AP2/ERF is a large family of TFs in plants, encoding transcriptional regulators with a variety of functions in the control of developmental and physiological processes including the integration of JA and ET signals [33]. The AP2/ERF family is classified into subfamilies containing AP2, DREB, EREBP and RAV TFs. Three AP2-like, two EREBP and two RAV TFs were rapidly up-regulated after 18:3-Glu elicitation (Additional file 2), suggesting that this family of TF may also play important roles in the orchestration of some of the plant's responses to insect feeding.

Two UniTags corresponding to the CCR4-associated factor 1 (CAF1) were up-regulated by 18:3-Glu elicitation. CAF1 is a subunit of the CCR4-NOT complex involved in mRNA degradation and Arabidopsis plants mutated in *CAF1a *and *b *genes are more susceptible to *Pseudomonas syringae *infection [34]. These authors hypothesized that the CAF1-containing complex controls the expression of a repressor of defense genes during pathogenesis. Our results suggested that the CCR4-NOT complex may also plays a role in defense responses against insects.

Several UniTags corresponding to putative cell wall-associated protein kinases (WAKs) were rapidly up-regulated after 18:3-Glu elicitation (Additional file 2). WAKs are transmembrane proteins containing a cytoplasmic Ser/Thr kinase domain and an extracellular domain in contact with components of the plant cell walls [35]. WAKs play important roles in cell expansion, pathogen resistance, and heavy-metal stress tolerance [36,37]. These protein kinases may associate changes in the cell wall structure after insect attack with downstream responses. Indirect evidence for rapid changes in cell wall structure and metabolism comes from the substantial number of genes associated with these processes that were up-regulated after 18:3-Glu elicitation (including an arabinogalactan protein (9-fold), beta-glucan-binding protein (9-fold), cellulose synthase (6-fold), α-expansin (2.5-fold), cell wall peroxidase (7-fold), raffinose synthase (7-fold), xyloglucan endotransglycosylases (3-fold), UDP-GlcUA 4-epimerase (3-fold) and xylose isomerase (4-fold; Additional file 2). Changes in the cell wall structure trigger JA- and ET-mediated defense responses in Arabidopsis as evidenced by the *cev1 *mutant, carrying a genetic lesion in a cellulose synthase gene [38]. Thus, changes in cell wall structure or homeostasis after mechanical damage and FAC elicitation might influence defensive signaling in a manner analogous to the *cev1 *mutant of Arabidopsis.

GSK3/SHAGGY-like kinase is a highly conserved Ser/Thr kinase involved in several signaling pathways. The Arabidopsis BRASSINOSTEROID-INSENSITIVE 2 (BIN2) gene encodes a GSK3/SHAGGY-like kinase and was identified as a negative regulator of brassinosteroid (BR) signaling [39]. Changes in the expression of transcripts for this kinase together with BRASSINOSTEROID INSENSITIVE 1-associated receptor kinase 1 (BAK1) suggested that BR play a role in the regulation of *M. sexta-N. attenuata *interaction. BR induces resistance against TMV, *P. syringae *and *Oidium *spp in tobacco plants [40]. Evidence for cytokinins also playing a role in this interaction came from the strong up-regulation of the *CYTOKONIN-REGULATED KINASE 1 *gene (*CRK1*) [41].

UniTags corresponding to PP2A and C were up-regulated while one UniTag corresponding to a PP2C isoform was down-regulated (Additional file 2). These regulators are known to play a central role in the control of defense-associated mechanisms. For example, the Arabidopsis AP2C1 (a PP2C) inactivates MPK4 and MPK6 and the mutant *ap2c1 *produces higher amounts of JA after wounding and is more resistant to phytophagous mites than WT. In contrast, plants with increased AP2C1 activity produced less ET and had compromised immunity against necrotrophic pathogens [42].

Among components associated to signal transduction processes, genes encoding for "Avr9-Cf9 rapidly elicited proteins" were the most predominant. The GO molecular function associated with these proteins was either protein kinase or receptor activity (Additional file 2) and some may correspond to R (resistance) genes in *N. attenuata*. The Avr9-Cf9 elicitor-receptor system is involved in the race-specific resistance of tomato (*S. lycopersicum*) against *Cladosporium fulvum *[43]. Interestingly, similar to wounding and OS elicitation, the Cf9 receptor induces the activation of WIPK and SIPK upon Avr9 binding and it has been proposed that these protein kinases are hubs in the integration of signals for diverse elicitors [44]. Thus, "Avr9/Cf9 rapidly elicited proteins" may be either important components in defense responses against insects or they may reflect redundancy (via activation of WIPK and SIPK and downstream gene expression) in the signal transduction pathway activated by wounding and FACs. An additional regulator that shows high homology to protein kinase receptors and R genes is the putative Hs1^pro-1^-like receptor (Additional file 2). The Hs1^pro-1 ^gene confers resistance to the beet cyst nematode *Heterodera schachtii *in sugar beet (*Beta vulgaris *L.) [45]. The transcript of Hs1^pro-1 ^is present at low levels in uninfected roots and it is induced specifically after nematode infection independently of SA, JA, ABA or wounding [46]. Its induction by 18:3-Glu in leaves on *N. attenuata *suggested that this gene may play a role in some plant responses against lepidopteran larvae in aboveground tissue. Up-regulation of UniTags corresponding to SGT1, a component of the Skp1-Cullin-F-box protein (SCF) ubiquitin ligases previously linked to early plant defenses responses conferred by R genes [47], also suggested that signaling pathways connected to R genes may be induced by insect elicitors in *N. attenuata*.

### Early changes in gene expression after 18:3-Glu elicitation reflect a rapid reprogramming of leaf metabolism

As a validation of the SuperSAGE approach, several UniTags identified as differentially regulated by 18:3-Glu elicitation corresponded to transcripts previously identified as differentially regulated by *M. sexta *larval feeding or OS/FAC elicitation [3,6,26]. Most of these genes corresponded to proteins involved in primary metabolism (Additional file 4). Changes in the expression of these genes supported the idea of a shift in primary metabolism to supply energy, C skeletons and reducing power for the synthesis of defensive compounds and to induce tolerance mechanisms [5]. Most of the putative regulatory factors identified in the present study were not identified in previous studies except for a WRKY transcription factor [26].

Changes occurring at the level of metabolism were among the most prevalent comprising more than 60 annotated UniTags. The up-regulation of twelve UniTags corresponding to transcripts for ribosomal structural proteins, four to translation initiation factors, two to tRNA synthases and several corresponding to amino acid biosynthesis (including tryptophan synthase, threonine deaminase, prephenate dehydrataseand 2-isopropylmalate synthase (Additional files 2 and 5) suggested that, within 30 min, 18:3-Glu elicitation stimulated an increased capacity for protein biosynthesis. Additionally, several annotated UniTags corresponding to genes involved in the generation of energy and C skeletons (e.g., subunits of ATP synthases, glyceraldehyde-3-phosphate-dehydrogenase, aldose-1-epimerase, fructose-bisphosphate aldolase, phosphofructokinase, sucrose synthase) and in the generation and metabolism of reducing power (e.g., NADP-dependent malic enzyme, Fed-NAD(P)^+ ^reductase, NAD kinase, nicotinamidase) showed strong up-regulations (Additional file 2). NAD-dependent malic enzymes (MEs) catalyze the oxidative decarboxylation of malate to produce pyruvate, CO_2_, and NADH in mitochondria [48] while NADP-dependent plastidic and cytosolic isoforms provide C skeletons and reducing power for defense responses, lignin biosynthesis and reactive oxygen species formation [49,50]. Changes in the expression of these genes are probably necessary to meet large requirements for NADPH to supply anabolic processes.

Interestingly, several UniTags corresponding to low abundant mRNAs for isoforms of photosynthetic proteins were found up-regulated between ~3 and 7-fold, including an oxygen-evolving protein, ribulose bisphosphate carboxylase activase, ribulose-1,5-bisphosphate carboxylase/oxygenase (RuBisCO) large subunit, chlorophyll a/b-binding protein, ferredoxin (Fed)-NADP(+) reductase and a PSI-H precursor (Additional files 2 and 5). In contrast to the up-regulation of these low abundant UniTags, high abundant UniTags corresponding to similar genes did not change significantly within 30 min after 18:3-Glu elicitation (e.g., Tags-4671, -3383, -1319 and -1133: photosystem II subunits; Tag-10791: light-harvesting chlorophyll a/b binding protein; Tags-11115, -11779, and -10899: RuBisCO subunits; Additional file 1). Why these low abundant isoforms of photosynthetic genes are up-regulated after elicitation is unknown. One possibility is that they play some specific roles during defense responses. In previous studies, it has been observed that after lepidopteran larvae feeding or OS/FAC elicitation transcripts encoding for photosynthetic enzymes (e.g., PSII, RuBisCO, RuBisCO activase) in attacked *N. attenuata *leaves tend to be down-regulated, with the lowest expression after several hours [6]. Long-term reductions in the synthesis of these proteins have been proposed as a mechanism that attacked plants use to reinvest resources into other processes such as the synthesis of secondary defense pathways or tolerance [5]. Within 30 min of FAC elicitation there were no significant reductions in the copy number of high abundance UniTags corresponding to mRNAs for photosynthetic proteins, suggesting that repressive mechanisms of major leaf isoforms act later during the FAC-induced response.

### Identification of two mediators of *M. sexta*-*N. attenuata *interaction

The analysis of six candidate genes by VIGS identified two putative regulatory components of resistance mechanisms against lepidopteran larval feeding. Caterpillars that fed for two weeks on plants silenced in the expression of a putative lipid phosphate phosphatase (LLP) and a protein of unknown function (UnkA) gained ~2-fold and ~1.3-fold more mass, respectively, than larvae grown on EV control plants (Figure 4). Gain of mass by the larvae can be achieved by increased foliar consumption by stimulatory mechanisms, increased efficiency of food intake by increased food quality, or a combination both [51]. By which mechanism *M. sexta *larvae grew larger on these plants is at present unknown and the subject of future investigations using stable transformed plants silenced in the expression of LPP and UnkA by RNA interference (RNAi). LPPs are signal transduction components that utilize a variety of lipid phosphate substrates including phosphatidic acid (PA), diacylglycerol pyrophosphate (DGPP), lyso-PA, ceramide 1-phosphate, and sphingosine 1-phosphate and it has been proposed that their function is to attenuate the signaling functions of these molecules [52,53].

Both LPP- and UnkA-silenced plants accumulated similar levels of JA and JA-Ile after wounding and FAC elicitation (Figure 5b), indicating that the effects of LLP and UnkA on *M. sexta *caterpillar performance was not the result of impaired JA biosynthesis. Together, these results suggested that mechanisms acting independently of JA biosynthesis must be affected in these plants to confer reduced resistance to *M. sexta *larval performance.

## Conclusions

The analysis of FAC-elicited *N. attenuata *plants by combined SuperSAGE and NGS enabled the identification of multiple factors with potential regulatory activity during the *M. sexta-N. attenuata *interaction. Together with the use of VIGS to analyze candidate gene function we provided experimental evidence for the participation of two of these potential regulators in this interaction. The further characterization of genetically stable LLP- and UnkA-silenced plants in addition to the identification and characterization of novel regulators based on the SuperSAGE data will shed light on mechanisms used by plants to control a large reorganization of their metabolism and physiology to adjust defense and tolerance mechanisms with growth and reproduction.

## Methods

### Plant growth and treatments

Seeds of the 30^th ^generation of an inbred line of *N. attenuata *plants were used as the wild-type genotype (WT) in all experiments. Plants were grown in the glasshouse at 26-30°C under 16 h of light. The second fully expanded leaf of rosette stage plants [54] were wounded by rolling a fabric pattern wheel three times on each side of the midvein and the wounds were immediately supplied with either 20 μL of 0.01% (v/v) Tween-20/water (solvent control) or 10 μL of synthetic *N*-linolenoyl-glutamic acid (18:3-Glu; 0.03 nmol/μL in 0.01% (v/v) Tween-20/water). Tissue was collected after 30 min of the treatments and frozen in liquid nitrogen for subsequent SuperSAGE analysis. For RT-qPCR experiments, leaf samples were harvested at different time points and frozen in liquid nitrogen for subsequent RNA extraction. For VIGS experiments, plants were grown in climate chambers under 20°C, 16 h light (1000 μmol m^-2^s^-1^) and 65% humidity. Wounding and FAC elicitation was performed as described above. Larvae of *M. sexta *were hatched overnight at 28°C and one neonate was placed on each VIGS-silenced plant (*n *= 32). The larval mass was measured using a microbalance after 4, 7, 11 and 15 days of the start of the experiment.

### RNA isolation and construction of SuperSAGE libraries

Total RNA was extracted by the phenol/chloroform-LiCl method as previously described [55] and the samples were cleaned using the Plant RNA Extraction Kit (Qiagen, Hilden, Germany) following commercial instructions. poly(A)-RNA was purified from total RNA with the Oligotex mRNA mini Kit (Qiagen) according to commercial instructions. Subsequent steps for the construction of the SuperSAGE libraries and 454 sequencing were performed as previously described [21,22]. To avoid methodological artifacts and to assure the detection of true transcript variants in the libraries, double *Nla*III digestions were performed during library generation [22].

For each library, 26 bp long tags were extracted from the sequences using the GXP-Tag sorter software provided by GenXPro GmbH (Frankfurt am Main, Germany). Library comparisons were carried out using the DiscoverySpace 4.01 software [Canada's Michael Smith Genome Sciences Centre, available at http://www.bcgsc.ca/discoveryspace]. Statistical analysis of differentially expressed tags was calculated according to [25]. Briefly, the probability distribution represented by equation (2) in [25] was used considering N_1 _and N_2 _as the total number of Tags in libraries 1 and 2, respectively, and *x *as the number of copies of a given Tag in library 1 and *y *as the number of copies of the same Tag in library 2. For fold-change (FC) calculations the libraries were normalized to 100,000 tags (Additional file 1) and the FC for each tag was calculated by dividing the number of tags in the normalized FAC library (F) by the number of tags in the normalized wounded (W) library (F vs. W). Tags absent in one of the libraries (tag count = 0) were set to 1 for calculation.

### Sequence homology alignments

BLAST searches were carried out using the BLASTN algorithm with the 12,774 UniTag sequences against plant nucleotide databases in GenBank (Additional file 1), filtering by selecting the *Nicotiana *(taxid: 4085) and Viridiplantae (taxid:33090) taxa. Low complexity regions were rejected and gap costs were set to 5-2. The sequence annotation presented in Additional file 1 was restricted to hits that matched a minimum of 20 nt. UniTags that matched to < 20 nt were annotated as "no hit" in this Table. Multiple entries separated by "or" were included when the UniTag matched more than one hit with identical scores. UniTags that matched to the negative strand of the nucleotide sequence in the database carry the prefix "minus" in their respective gene identification (GI) column (Additional file 1). For protein and gene ontology (GO) annotations of differentially expressed UniTags (Additional file 2), their respective nucleotide sequences (Additional file 1) were used to perform BLASTX against the NCBI non-redundant protein database and hits with scores < 9.10^-4 ^were used for GO determination based on the UniProtKB/TrEMBL protein databases.

### Rapid amplification of cDNA ends (3'-RACE)

For cDNA synthesis, the 3'RACE System for Rapid Amplification of cDNA Ends (Invitrogen, Karlsruhe, Germany) was used following the manufacturer's instructions and using UniTag- and gene-specific primers (Additional file 5). The PCR products were cloned into the pGEM-T easy vector (Promega, Madison, WI) and sequenced using universal primers.

### Real time quantitative PCR

To analyze the mRNA levels corresponding to the 27 selected UniTags (Table 3), rosette stage *N.attenuata *WT plants were either wounded or FAC elicited as described above. Leaves were harvested at 0, 30, 60, 90, and 120 min after the treatments, total RNA extracted using the TRIzol^® ^reagent (Invitrogen) and DNase-I treated (Fermentas, St. Leon-Rot, Germany). Five μg of total RNA were reverse transcribed using oligo(dT)18 and SuperScript reverse transcriptase II (Invitrogen). Quantitative real-time PCR (qPCR) was performed with a Mx3005P Multiplex qPCR system (Stratagene, La Jolla, CA) and the qPCR Core kit for SYBR^® ^Green I (Eurogentec, Liege, Belgium). Relative quantification of mRNA levels was performed by the comparative ΔCt method using the eukaryotic elongation factor 1A (NaeEF1A) mRNA as an internal standard. The sequences of the primers used for qPCR are listed in Additional file 5. All the reactions were performed with three biological replicates.

### Virus induced gene silencing

Virus-induced gene silencing (VIGS) based on the tobacco rattle virus (TRV) was used to transiently silence the genes listed in Table 4 in *N. attenuata *as previously described [56]. The accession numbers corresponding to the sequences are listed under "Accession numbers" below. Fragments of ~300 bp were amplified by PCR with the specific primers listed in Additional file 5. PCR products were digested with BamH1 and Sal1 and inserted into plasmid pTV00 in antisense orientation. Plants transformed with the empty vector (EV) were used as control. Plants were analyzed after 15 days of leaf infiltration. Efficiency of gene silencing was evaluated by qPCR after 1 h of 18:3-Glu elicitation using the primers listed in Additional file 5.

### Phytohormone extraction and quantification

For analysis of JA and JA-Ile, 0.1 g of frozen leaf material was homogenized in FastPrep^® ^tubes containing 1 g of FastPrep^® ^matrix (Bio101, La Jolla, CA) and 1 mL ethylacetate spiked with 0.1 μg of [9,10-^2^H_2_]-dihydro-JA and [^13^C_6_]-JA-Ile. Homogenates were centrifuged for 10 min at 4°C, the organic phase collected and plant material re-extracted with 0.5 mL ethylacetate. Organic phases were combined and the samples evaporated to dryness. The dry residue was reconstituted in 0.2 mL of 70% (v/v) methanol/water for analysis on a LC-(ESI)-MS/MS system as previously described [57].

### Statistical analysis

Repeated-measurement and univariate ANOVA were calculated using SPSS v. 17.0.

### Accession numbers

The SuperSAGE data was deposited in the Gene Expression Omnibus (GEO) public domain under the accession [GenBank: GSE18595]. The accession numbers for ESTs corresponding to the extended Unitag sequences are Tag-129: [GenBank: GT184388]; Tag-837: [GenBank: GT184389]; Tag-895: [GenBank: GT184390]; Tag-995: [GenBank: GT184391]; Tag-1439: [GenBank: GT184392]; Tag-1844: [GenBank: GT184393]; Tag-2067: [GenBank: GT184394]; Tag-2452: [GenBank: GT184395]; Tag-2815: [GenBank: GT184396]; Tag-2978: [GenBank: GT184397]; Tag-2990: [GenBank: GT184398]; Tag-4898: [GenBank: GT184399]; Tag-5283: [GenBank: GT184400]; Tag-5869: [GenBank: GT184401]; Tag-6032: [GenBank: GT184402]; Tag-6199: [GenBank: GT184403]; Tag-6205: [GenBank: GT184404]; Tag-6642: [GenBank: GT184405]; Tag-6938: [GenBank: GT184406]; Tag-7036: [GenBank: GT184407]; Tag-7795: [GenBank: GT184408]; Tag-9719: [GenBank: GT184409]; Tag-10039: [GenBank: GT184410]; Tag-11166: [GenBank: GT184411]; Tag-11559: [GenBank: GT184412]; Tag-12314: [GenBank: GT184413].

## Authors' contributions

PG and GB carried out the experiments, analyzed the data and drafted the manuscript. RJ and BR carried out experiments and analyzed the data. SS analyzed the data. ITB participated in the design and coordination of the study and helped to draft the manuscript. GB conceived of the study, participated in its design and coordination and helped to draft the manuscript. All authors read and approved the final manuscript.

## Supplementary Material

Additional file 1 Complete list of UniTag sequences, copy numbers, and annotations to GenBank nucleotide databases. Click here for file

Additional file 2 Complete list of differentially expressed UniTags and GO categorization. Click here for file

Additional file 3 **Supplementary Figures.** Figure S1. Analysis of mRNA accumulation corresponding to 12 UniTags by qPCR. Figure S2. *M.sexta *larval performance on VIGS silenced plants. Click here for file

Additional file 4 List of differentially expressed UniTags previously identified by differential expression techniques. Click here for file

Additional file 5 **Supplementary Tables**. Table S1. Primers for elongation of cDNAs correspondig to UniTags. Table S2. Primers for qPCR. Table S3. Primers for VIGS analysis. Click here for file
