# Supplementary material for: SuperSAGE analysis of the Nicotiana attenuata transcriptome after fatty acid-amino acid elicitation (FAC): identification of early mediators of insect responses
Source: BMC Plant Biol. 2010 Apr 14;10:66. doi: 10.1186/1471-2229-10-66 (PMC3095340; doi:10.1186/1471-2229-10-66)
Supplement: Additional file 3 — Supplementary Figures. Figure S1. Analysis of mRNA accumulation corresponding to 12 UniTags by qPCR. Figure S2. M.sexta larval performance on VIGS silenced plants. [file 1471-2229-10-66-S3.PDF]

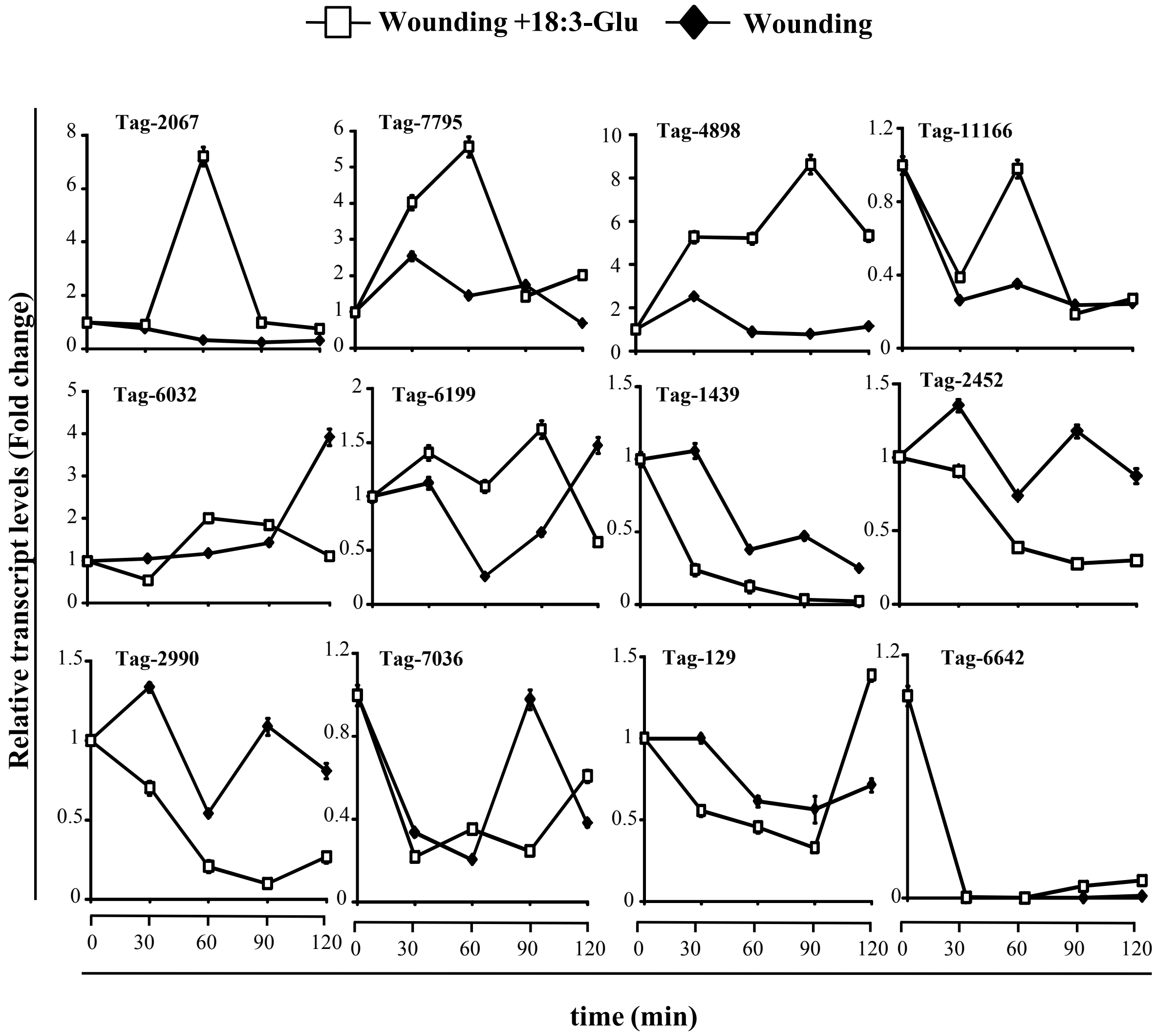

Figure S1. Analysis of mRNA accumulation corresponding to selected UniTags by qPCR. Kinetics of induction of mRNAs for 12 UniTags analyzed by qPCR after wounding and 18:3-Glu elicitation . Relative mRNA quantification was performed using the eEF1A as a reference gene for normalization and the data is expressed as fold-change relative to time 0 (unelicited leaves). Values at this time point were set arbitrary to 1. Transcripts levels were analyzed in three biological replicates (*n*=3).

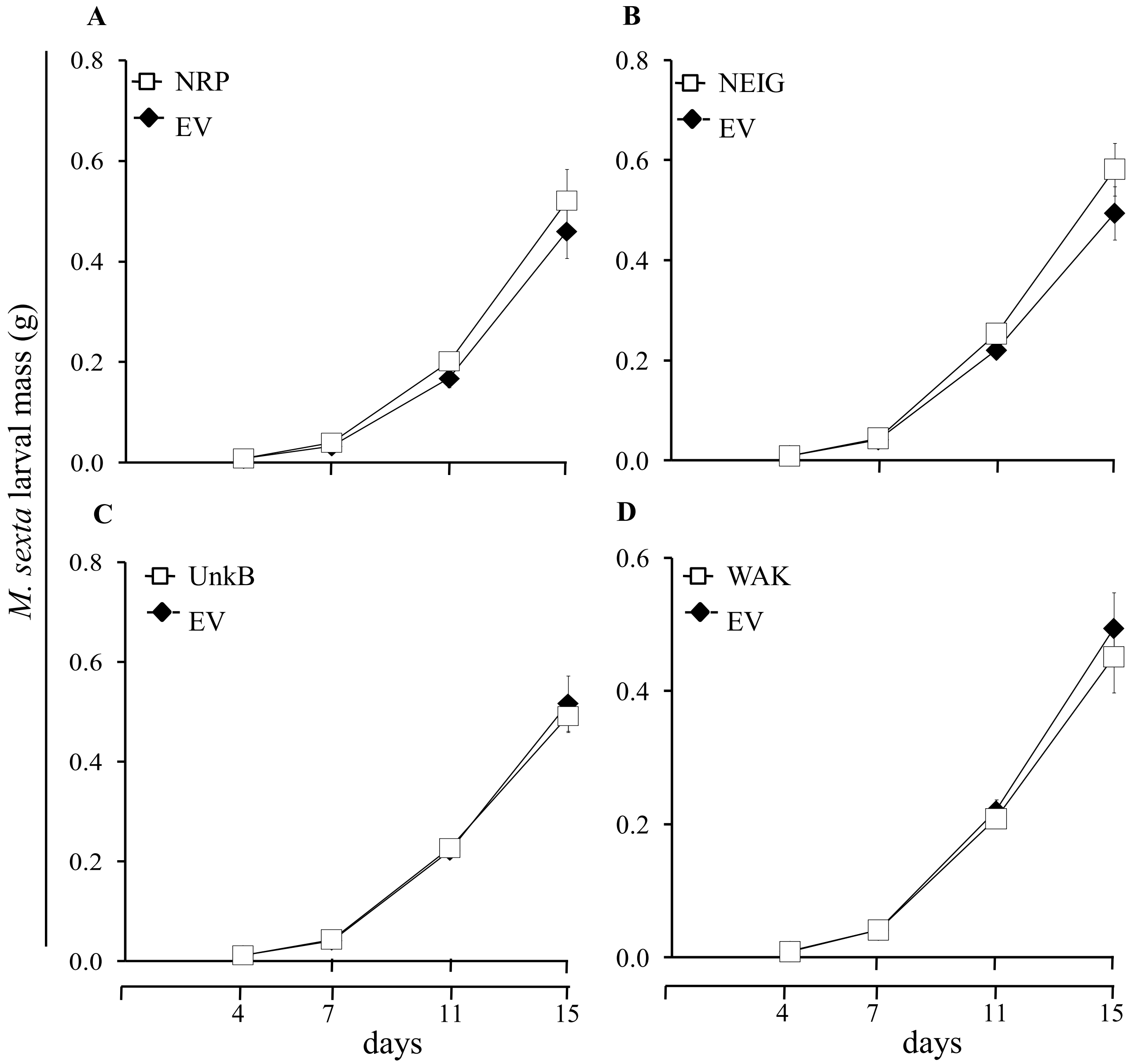

Figure S2. *M.sexta* larval performance on VIGS silenced plants.

N.attenuata plants were silenced in the expression of (A) NRP, (B) NEIG, (C) UnkB and (D) WAK by VIGS. Plants transformed with the empty vector (EV) were used as controls. Results are presented as the mean (+/- SE) of *M.sexta* larval mass after 4, 7, 11 and 15 days of feeding on EV and silenced plants (*n*=32 for each genotype). Statistical analysis was performed by repeated-measurement ANOVA and no significant differences were observed.
