# Supplementary material for: SuperSAGE analysis of the Nicotiana attenuata transcriptome after fatty acid-amino acid elicitation (FAC): identification of early mediators of insect responses
Source: BMC Plant Biol. 2010 Apr 14;10:66. doi: 10.1186/1471-2229-10-66 (PMC3095340; doi:10.1186/1471-2229-10-66)
Supplement: Additional file 4 — List of differentially expressed UniTags previously identified by differential expression techniques. [file 1471-2229-10-66-S4.PDF]

Table S1. Primers used for elongation of cDNAs correspondig to UniTags.

Nested primers for 3'RACE

Tag 837_A	CATGAATCATCCAATATGGTATG
Tag 837_B	GAATCATCCAATATGGTATGGGC
Tag 895_A	CATGAATGACACTAATGAATTCG
Tag 895_B	GAATGACACTAATGAATTCGTCG
Tag 1439_A	CATGACTGCTGTCAGACGAACTG
Tag 1439_B	GACTGCTGTCAGACGAACTGCAC
Tag 2452_A	CATGATGAATACGAGCAGCTTCG
Tag 2452_B	GATGAATACGAGCAGCTTCGGGT
Tag 2978_A	CATGATTTTTTTTCCTTCTGCTG
Tag 2978_B	GATTTTTTTTCCTTCTGCTGTAT
Tag 2990_A	CATGCAAAACGTACACCGAGAAA
Tag 2990_B	GCAAAACGTACACCGAGAAAGAA
Tag 4898_A	CATGCTGCTGGGACTCTCGTA
Tag 4898_B	TGCTGGGACTCTCGTATACAG
Tag 6199_A	CATGGATCGGCAAACAAAGAGAT
Tag 6199_B	GGATCGGCAAACAAAGAGATTAT
Tag 6642_A	CATGGCCAAGAGTACGTTCTC
Tag 6642_B	CCAAGAGTACGTTCTCAAAGG
Tag 11166_A	CATGTGTCAAGCTGGAAAACT
Tag 11166_B	GTCAAGCTGGAAAACTTGCCA
Tag 12314_A	CATGTTTAGAGCAATGAGTACAC
Tag 12314_B	GTTTAGAGCAATGAGTACACGAA

Specific primers

Tag 129_Fw	TCTTAGCGTTCCATCCTCAAGA
Tag 129_Rev	ACATGGTTGCTAGTGGTCGAAG
Tag 995 _Fw	TTGGCTTGTTTCTTGAATTCATG
Tag 995_Rv	CAATTCTCAAATGGACTGATTCA
Tag 1844_Fw	GAACATGATAAGAAGAATTTGATGA
Tag 1844_Rv	GAAGACGCTCATATATATGGTAC
Tag 2067_Fw	GCAGTCAAGTAGGTCTACTAC
Tag 2067_Rv	CATGAGTTGGTGGATTCAAATC
Tag 2815_Fw	CAAGTTGCTCTTGGGGAGATCA
Tag 2815_Rv	CAGCTCCACTGCTTTGCAACTC
Tag 5283_Fw	CATTCGATAACCCCAGATTTTTC
Tag 5283_Rv	TGCTTGAATTTCACTCGCAGC
Tag 5869_Fw	GCTGAGAAATGTAATGACTTCTG
Tag 5869_Rv	AAAACTTAACTTGCAAAGTCTCC
Tag 6032_Fw	CATGGAGGTCTTTCTCGTTATC
Tag 6032_Rv	GAACGCAAAGGTTTAAAACGTATA
Tag 6205_Fw	CTGATCCTAGACCGTATGCG
Tag 6205_Rv	AAGCATTTTTGACGCGTAGATCC
Tag 6938_Fw	TCATGGCTCGGATTTGCATCTC
Tag 6938_Rv	GATCAATCGAGGTGGAGAACTC
Tag 7036_Fw	TGAAACTCGTGCTGAGATCGT
Tag 7036_Rv	ATCCAGGTAAGTTGTCAGCAG
Tag 7795_Fw	CATGGGTTATTCAGTGCTGT
Tag 7795_Rv	AGCATAGACCAAAGCACCATTG
Tag 9434_Fw	AGTGTGATGATAATTCAACTGCAGC
Tag 9434_Rv	AGTTGAGAAAGAATTCATCAGGTCAC
Tag 9719_Fw	TGATGTCTATTTTCAGTTCCTTTG
Tag 9719_Rv	TTCCTGAATTTACAGCAGAATAC
Tag 10039_Fw	CTTGGGTTTCTATCATTGTATTTG
Tag 10039-Rv	CCGTTAGTATGGTGGACATG
Tag 11559_Fw	GCTAATACTAGCTGTGGTAGAT
Tag 11559_Rv	CATGCATGTTATCAGTTAACTAA

Table S2. Primers for qRT-PCR.

Tag 129_qFw	TAGCCATTAGCATGGAGGATTCAAC	Tag 6032_qFw	CATGGAGGTCTTTCTCGTTATCTGA
Tag 129_qRv	TCTTGTCCCATGTATCAATGCTCCA	Tag 6032_qRv	TTACAATGACAGAGAGCCAGACTCA
Tag 837_qFw	GAATCATCCAATATGGTATGGGCAA	Tag 6199_qFw	AGGAACGGACTGCACAAGATCATTC
Tag 837_qRv	TGATGACGAGAGCCATCAGCCCTT	Tag 6199_qRv	TGGATCGGCAAACAAAGAGATTATTC
Tag 895_qFw	TGAATTCGTCGTTAAGCCTTCGAGT	Tag 6205_qFw	CTGATCCTAGACCGTATGCGAACA
Tag 895_qRv	CTGTTAGTCGTAGTCGAATTCGGAT	Tag 6205_qRv	GCTGACAACCGTCGTCTCATCAGA
Tag 995_qFw	GGCTTGTTTCTTGAATTCATGTTTCT	Tag 6642_qFw	CCAAGAGTACGTTCTCAAAGGATCA
Tag 995_qRv	CAAGTCAGAGTTAGTGCAGAGAATG	Tag 6642_qRv	TTGTCCTCTGCTCAGCAGCTCCGT
Tag 1439 _qFw	ACTGCTGTCAGACGAACTGCACAGTT	Tag 6938_qFw	CAATCGAGGTGGAGAACTCAAAACG
Tag 1439_qRv	TTCCTCCCTGCTTAATGGCTTTTTG	Tag 6938_qRv	AGGCTGAGCCGACCAGTGAGAATC
Tag 1844_qFw	GTCATTACATGGTCATCCTAAGAAG	Tag 7036_qRv	GCAAAAGTCTCAGAATCGATGGTCT
Tag 1844_qRv	CGTACACAACCAGAGTGATACATAA	Tag 7036_qFw	GAAACTCGTGCTGAGATCGTTGAAC
Tag 2067_qFw	AGTCAAGTAGGTCTACTACTCTATG	Tag 7795_qFw	AGCATAGACCAAAGCACCATTGTCC
Tag 2067_qRv	GAGCACCTGCTATGTTGGTTGCCA	Tag 7795_qRv	TTGCACTATGCAGCCTATTATGGTC
Tag 2452_qFw	GATGAATACGAGCAGCTTCGGGTAA	Tag 9719_qFw	CTTGTATCAGATCACAAGACTGGTG
Tag 2452_qRv	TCGATCCAATGTCATGTGCATTGAG	Tag 9719_qRv	TAGGGAATGATAGTCTCGAAACAGT
Tag 2815_qFw	AGCTCCACTGCTTTGCAACTCAATC	Tag 10039_qFw	CACGTGTGGATGACTACTGGCATC
Tag 2815_qRv	TCTGATTCTTTGCTTGAGGCTCAGT	Tag 10039_qRv	CGTTAGTATGGTGGACATGTTGT
Tag 2978_qFw	TTCTGCTGTATAAGTTTAATTGTCCA	Tag 11166_qFw	GTCAAGCTGGAAAACTTGCCAGTG
Tag 2978_qRv	ACAAGTTATATCCGTAAATACCACCA	Tag 11166_qRv	CATTTTTCATTCGGTCTCATAGGAC
Tag 2990_qFw	CGTACACCGAGAAAGAAGGCCTCA	Tag 11559_qFw	GTCCTTGTTGAGCTGATATCATCAC
Tag 2990_qRv	AGTTTCTGTTGCCAGATGCCAAGAG	Tag 11559_qRv	AGCTAGCTCTGCAACTGAAGTAGTC
Tag 4898_qFw	ATCTGCATCGTCGAGTAGTAGACAC	Tag 12314 _qFw	CCAATCTTGGGTAAGCCAAAGTTGA
Tag 4898_qRv	CGCTCTACAATTATCACAAATCACTC	Tag 12314 _qRv	ACTGAGTAGTCGCTCGGAAATTCTC
Tag 5283_qFw	TCATGAACACAAGCTAATGCACTTG	Tag 9434_qFw	AGTGTGATGATAATTCAACTGCAGC
Tag 5283_qRv	GTTCAGTCCTTGAAATACAGTAGACT	Tag 9434_qRv	AGTTGAGAAAGAATTCATCAGGTCAC
Tag 5869_qFw	GCTTGATCTGAGTTAACATACACCT		
Tag 5869_qRv	TGCTGAGAAATGTAATGACTTCTGAG		

Table S3. Primers used for VIGS.

Primers used for construction of VIGS vectors.

EIG_Fw	GCGGCGGTCGACTTGCATCATCTGGGAAGAGG
EIG_Rv	GCGGCGGGATCCGCATAGGTCTGGCAAGTACAC
LPP_Fw	GCGGCGGTCGACATTGTATTTGGCAGGGAAG
LPP_Rv	GCGGCGGGATCCGTTAGTATGGTGGACATG
NRP_Fw	GCGGCGGTCGACACGACGGTTGTCAGCCAGAC
NRP_Rv	GCGGCGGGATCCGTAGATCCATGTTTCGAGGATC
UNKA_Fw	GCGGCGGTCGACAAACTGTGTCACCTACAGGG
UNKA_Rv	GCGGCGGGATCCGGTCTCTACAGCTTGTATGC
UNKB_Fw	GCGGCGGTCGACAGGAGCATTCTGCTCCAATC
UNKB_Rv	GCGGCGGGATCCTGCTTCCATGTTGGATTTGG
WAK_Fw	GCGGCGGTCGACAGCGATGTGTATAGCTTCGG
WAK_Rv	GCGGCGGGATCCTCTGGTTTTCAAACTCGCCC

Primers used to evaluate gene silencing ficiency in VIGS-silenced plants

EIG_qFw	TGCCAACAAATGTTTGTGCATTCTC
EIG_qRv	GTGTGGGTTGCTCCTCTGCTTC
LPP_qFw	CTTGGGTTTCTATCATTGTATTTGGCA
LPP_qRv	TGATGCCAGTAGTCATCCACACGTG
NRP_qFw	CTGATCCTAGACCGTATGCGAACA
NRP_qRv	ACACCTCCTCATTTCGCATTCAATG
UnkA_qFw	TATGGTATGGGCAAGTAGAGATGAC
UnkA_qRv	CCTTAGTATTCATTCATCATTACCAC
UnkB_qFw	AAGGAAGAGGAGCATTCTGCTCC
UnkB_qRw	CACCGCAGAATCAAAATCACTTTAC
WAK_qFw	CATGCATGTTTCAGTTAACTAATA
WAK_qRv	TGACATTAATCTCTTCTTTCTTCTG
